# Use of a Visual Aid in addition to a Collector Bag to Evaluate Postpartum Blood loss: A Prospective Simulation Study

**DOI:** 10.1038/srep46333

**Published:** 2017-04-21

**Authors:** M. Brooks, G. Legendre, S. Brun, P. -E. Bouet, L. Pereira Mendes, B. Merlot, L. Sentilhes

**Affiliations:** 1 Department of Obstetrics and Gynecology, Angers University Hospital, Angers, France; 2 Department of Obstetrics and Gynecology, Bordeaux University Hospital, Bordeaux, France

## Abstract

Postpartum hemorrhage (PPH) is one of the most common causes of mortality in obstetrics worldwide. The accuracy of estimated blood loss is a priority in determining appropriate treatment. Will the additional use of a visual aid improve physicians’ accuracy in estimating blood loss compared to the use of a collector bag and baby scale alone? Simulation training sessions created three vaginal delivery scenarios for participants to estimate volumes of blood loss: firstly, using only a collector bag and a baby weight scale and secondly, adding a visual aid depicting known volumes of blood. The primary endpoint was to determine if participants could accurately evaluate blood loss within a 20% error margin. The addition of the visual estimator resulted in overestimation of blood loss. The rates of participants’ estimations were significantly more accurate when using the collector bag with the baby weight scale without the addition of the visual aid; 85.5% versus 33.3% (p < 0.01) for 350 mL, 88.4% versus 50.7% (p < 0.01) for 1100 mL and 88.4% versus 78.3% (p < 0.01) for 2500 mL, respectively. Additional use of a visual aid with a collector bag does not seem to be useful in improving the accuracy in the estimation of blood loss.

Postpartum Hemorrhage (PPH) occurs in 5% to 10% of all deliveries^1^ worldwide. It is the primary cause of morbidity and mortality in the obstetrical field as well as the first cause of admission into the intensive care unit for the postpartum woman^1^,^2^,^3^,^4^. Worldwide, 25% of maternal deaths are due to PPH^5^. According to the Royal College of Obstetricians and Gynaecologists (RCOG)^6^ and the most recent French guidelines released in 2014^1^,^4^, PPH is defined by a minimum of 500 ml of blood loss within the first 24 hours of delivery and is considered major when the blood loss is more than 1000 ml^3^,^4^,^6^. An inaccurate estimation of blood loss could result in a delay in treatment, which may negatively influence patient care^7^. In developed countries, at least 60% of the maternal post-natal deaths^8^ are considered related to suboptimal treatment because of a delay in PPH diagnosis, as well as a delay in management of the third stage of labor, and therefore could be avoided^4^.

The visual estimation of blood loss by professionals, without using a graduated collector bag, has been evaluated in several studies^9^,^10^,^11^,^12^ and has been shown to lack accuracy and to underestimate actual blood loss by up to 30%. The percent of inaccuracy also increases as the volume of blood loss increases^10^. The collector bag is an objective tool used to estimate blood loss with an accuracy of 90%^9^,^13^,^14^,^15^. It has been used several times in international randomized controlled clinical trials as the primary endpoint to objectively assess PPH^16^,^17^,^18^. All authorities base their PPH definition and threshold to intervene mainly on the amount of blood loss and underline that visual assessment of blood loss is unreliable and underestimates the true blood loss^19^. However, there is an apparent contradiction as most of them do not recommend any objective tools to be used to measure blood loss before a diagnosis of PPH although they do recommend these tools once the diagnosis is established^19^,^20^,^21^. For example, the Royal College of Obstetricians and Gynaecologists (RCOG)^6^ stated that “more accurate methods than visual blood loss may be used such as blood collection drapes for vaginal deliveries and weighing swabs” and that “written and pictorial guidelines may help staff working in labour wards to estimate blood loss”. Meanwhile, The French College of Gynecologists and Obstetricians (CNGOF)^4^ recommends the use of a collector bag only once the PPH diagnosis is established.

To help with PPH assessment, the International Federation of Gynecology and Obstetrics (FIGO) and the RCOG^6^ organized simulation training sessions such as masterclasses with workshops with the RCOG stating that “participating in clinical reconstructions may encourage early diagnosis and prompt treatment of PPH.”

A recent American study has suggested that a visual aid depicting known volumes of blood on obstetric materials can improve accuracy of blood volume estimation among obstetric providers^20^. This visual aid is therefore currently used by some obstetric care providers in their protocol for managing the third stage of labor as well as in training simulation sessions^6^,^20^. However, the use of a visual aid in association with a collector bag compared to the use of a collector bag and baby scale only in the estimation of postpartum blood loss has not yet been evaluated.

The aim of this study was to determine if the additional use of a visual aid could improve physicians’ accuracy in estimating blood loss compared to the use of a collector bag with baby scale only.

## Materials and Methods

A prospective study occurred in a third level maternity ward at a French teaching hospital from October 1^st^ 2013 to October 13^th^ 2013. This prospective study evaluated the accuracy of the obstetrics providers’ estimations of blood loss after a vaginal delivery during training simulation sessions designed to mimic clinical scenarios. All the medical staff, obstetricians and Gynecologists (OG), midwives, and residents in obstetrics and gynecology (OBGYN) were invited to participate. The participants chosen for this study were those that were most likely to be facing a PPH diagnostic situation in the delivery room. Participants volunteered to participate in the study and were aware of being tested on the estimation of blood loss. During both parts of the two part study, each participant’s estimation was compared to an actual volume of blood loss which was only known by the researchers. Each participant had to evaluate blood loss for three different volume scenarios for each part of the study. They consequently had six volumes to estimate. The three blood loss volumes were identical for both parts of the study and were 350 ml, 1100 ml and 2500 ml. However, the ordering of these volume estimating scenarios was randomly assigned by a computer program for both parts of the study resulting in varying sequencing of scenarios among participants. For each participant, the estimation of postpartum blood loss was made on the same day for both parts of the study. In the delivery room where the study occurred, participants had to estimate postpartum blood loss (in the graduated collector bag and on different obstetrical materials around the mannequin) within two minutes to recreate a more time sensitive clinical environment. The fluid used to recreate blood was in color and density similar to real blood. To determine participant characteristics, each of the participants was asked to fill out a questionnaire.

### First part of the study: use of a collector bag and baby scale to evaluate postpartum blood loss

**Figure 1:**
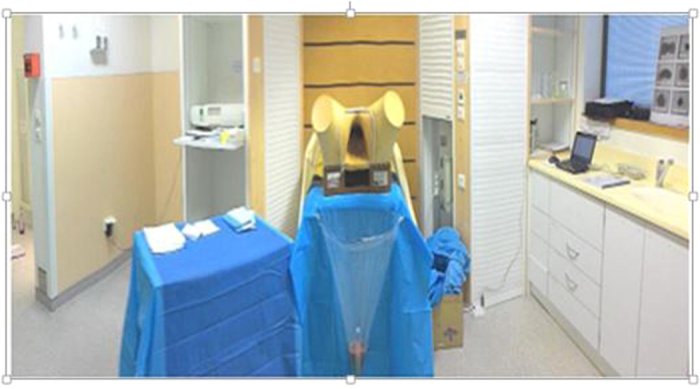
Scenario which recreated a vaginal delivery during the training session.

All three blood loss scenarios recreated a normal vaginal delivery. The training session is described as follows: in a delivery room, a mannequin was placed in the lithotomy position with a graduated collector bag directly underneath it *(Medline International France − 44110 Chateaubriand, FRANCE - reference DGFMA173)*, and the delivery equipment nearby. This collector bag is used routinely by our caregivers to estimate blood loss after each vaginal delivery in the lithotomy position, which is the current standard position for vaginal deliveries throughout France^7^. The collector bag as described in this study is a plastic collector bag graduated every 100 ml from zero ml to 1500 ml. Each volume was to be analyzed separately by each individual participant (Fig. 1). There were three volumes to be analyzed: 350 ml, 1100 ml and 2500 ml. This study was designed for participants to assume that the collector bag contained only blood, and that the fluid on the obstetrical equipment nearby was also blood. According to the study, blood loss was contained in the collector bag but also on obstetrics materials such as a kidney dish, hospital sheets, incontinence pad, and sanitary towel. All of the equipment’s weight (prior to the addition of blood) was known and presented on an accessible table in the same room where the simulation training occurred (sanitary towel, incontinence pad, hospital sheets and kidney dish). A baby weight scale was set up on the side to be used by participants if they wanted to weigh the obstetrical materials covered in blood for estimating the three different volumes.

### Second part of the study: use of a visual aid depicting known volumes of blood on obstetric materials to estimate blood loss in addition to a collector bag and a baby weight scale

**Figure 2:**
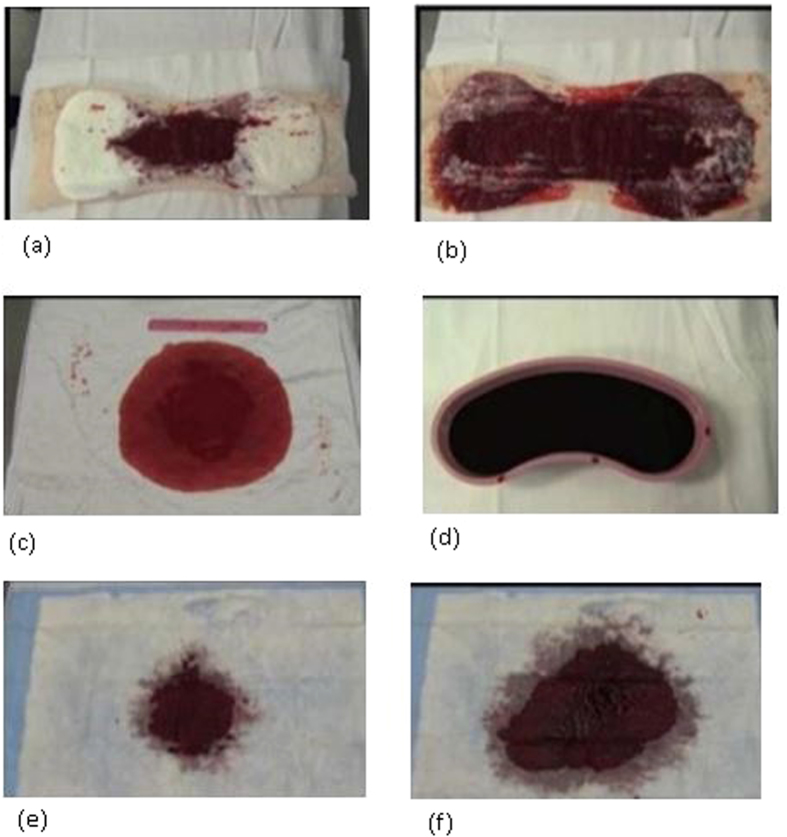
(**a–f**) visual aid depicting known volumes of blood on obstetric materials used during the second part of the study from Zuckerwise *et al*.^20^ (**a**) Soaked Sanitary Towel 100 ml. (**b**) Soaked Sanitary Towel 300 ml. (**c**) Hospital sheet 250 ml. (**d**) Full Kidney Dish 500 ml. (**e**) Incontinence pad 50 ml. (**f**) Incontinence pad 200 ml.

For this second part of the study, the delivery room and the clinical ambiance which recreated a vaginal delivery was identical to that described in the first part of the study above. Participants had to estimate postpartum blood loss for the same three volumes previously analyzed: 350 ml, 1100 ml, and 2500 ml. Estimation of postpartum blood loss during this second part was presumed to be similar to actual clinical PPH situations. Participants had to estimate blood loss while using a visual aid in addition to the identical collector bag and baby weight scale used in part one of the study (Fig. 2).

The visual aid used was a small pocket card containing images of blood loss on common obstetric materials to serve as a reference (Fig. 2). This visual aid has been described and used before in a British study, by Bose and al^22^ as well as in a recent American study, by Zuckerwise and al^20^. It is meant to be used to help in the estimation of blood loss when collected on several obstetrical materials such as an incontinence pad or kidney dish^20^. The trial design uses each participant as their own control (with or without the visual aid). Each participant could consult the visual aid as they were assessing the blood loss contained in the collector bag and on the several obstetrics materials.

### Statistical Analysis

The population qualitative parameters were described in numbers and percentage. The quantitative parameters, which were normally distributed, were described by median and standard deviation (SD) as well as interquartile range (IQR). During the first part of the study, for each of the three volumes which participants were asked to estimate, the professionals’ estimation of the blood loss was indicated as a mean of the difference between the estimated volume of blood loss (EBV) and the real volume of blood loss (RBV):[ (EBV-RBV)/RBV+/−SD]. The mean difference found for (EBV – RBV)/RBV is written as a percentage in order to compare all the estimations made on the different RBVs, based on a common unit. The Bland and Altman method^23^ was used to evaluate the accuracy in between EBV and RBV, using for the first part of the study the collector bag and baby scale and for the second part the visual aid in addition to the collector bag and baby scale. The Bland and Altman method is used to evaluate the agreement between two measurement techniques. In this situation, the Bland and Altman method was used to evaluate obstetrics care providers’ estimations of blood loss while using two different techniques to estimate blood loss in comparison to real blood loss whose volumes were previously known by the researchers. The first technique was the visual estimation of blood loss using a collector bag with a baby weight scale; the second technique was using a visual aid in addition to a collector bag. The limits of agreement were defined as the mean difference plus or minus 1.96SD of the differences (with a 95% confident interval). The differences between the two measurement techniques were plotted against the averages of the two techniques. For each of the three volume loss scenarios, a univariate analysis with a linear regression was used to evaluate if a dependent variable could explain the differences found between the participants’ responses. The Wilcoxon test was used to analyze data for each estimation of blood loss, with or without using a visual aid in addition to the collector bag. For each analysis p ≤ 0.05 was considered as significant.

The primary endpoint was to determine if participants could accurately evaluate blood loss, in each of the three scenarios, within a 20% margin of error. Estimations made that were more than 20% of the real volume of blood loss were considered overestimations, while estimations made that were less than 20% of the real volume of blood loss were considered underestimations^12^.

## Results

Sixty-nine professionals from the maternity ward were available to participate to the study (including fifty-one midwives, ten obstetricians, and eight residents in OBGYN).

Characteristics of the population of the study are described in Table 1.

**Figure 3:**
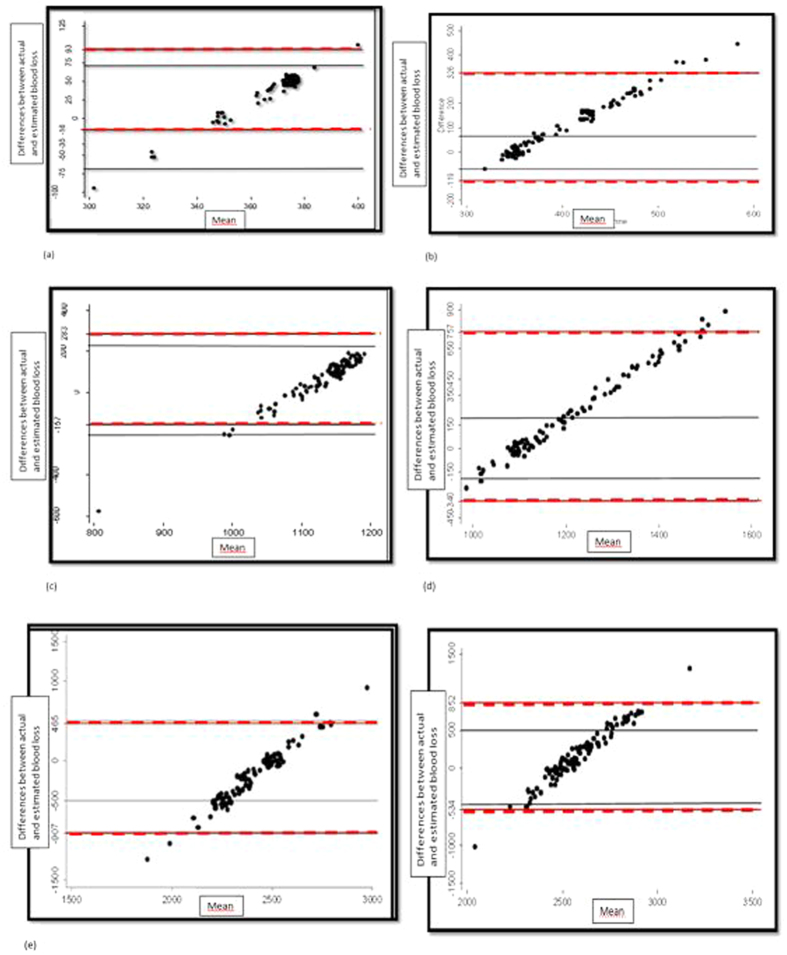
(**a–f**) Bland Altman chart for evaluation of postpartum blood loss with and without using the visual scale. (**a**) Bland Altman chart: Volume 350cc without visual aid. (**b**) Bland Altman chart: Volume 350cc: using a visual aid. (**c**) Bland Altman chart: Volume 1100cc without using a visual aid. (**d**) Bland Altman chart: Volume 1100cc using a visual aid. (**e**) Bland Altman chart: Volume 2500cc without using a visual aid. (**f**) Bland Altman chart: Volume 2500cc using a visual aid. (**a,c,e**) are without the visual aid for the volume 350cc, 1100cc, 2500cc respectively. (**b,d,f**) are using the visual aid in addition to the collector bag and the baby weight scale. The two dashed red lines correspond to the 20% error margin for each volume estimated. The two black lines correspond to the limits of agreement for each volume estimated (according to the Bland Altman method). The vertical axis represents the differences between the known and the estimated volume in estimation of blood loss. The horizontal axis represents the mean of estimation of estimated blood loss and the known volume of blood loss.

In all cases, the rates of participant estimations were significantly more accurate when using the collector bag with the baby weight scale without the addition of the visual aid; 85.5% versus 33.3% (p < 0.01) for 350 mL, 88.4% versus 50.7% (p < 0.01) for 1100 mL and 88.4% versus 78.3% (p < 0.01) for 2500 mL, respectively (Table 2). Figure 3a–f represent Bland Altman charts for each of the three scenarios (350 ml, 1100 ml, and 2500 ml) while using a visual scale in addition to the collector bag and while not using the visual scale.

The vertical axis represents the differences in estimation of blood loss and the horizontal axis represents the mean estimation of blood loss. Outcomes demonstrated that, while using a visual aid in addition to a collector bag, the limits of agreement determined by the Bland Altman method reference [−262–239] for each of the three volume analysed (350 ml, 1100 ml, 2500 ml) were outside each of their confidence intervals, and all closer to the +20% margin of acceptability. It demonstrated a tendency of professionals to overestimate blood loss while using the visual aid (Fig. 3b,d and f). Results for the use of the collector bag alone demonstrated that the limits of agreement, for each volume analysed, were mostly included in their confidence interval, however there was an underestimation tendency for the highest volume and an overestimation tendency for the lowest volume (Fig. 3a,c and e).

## Discussion

Our results suggest that the additional use of a visual aid with a collector bag and a baby weight scale results in an overestimation of blood loss and is consequently not associated with the improvement of the estimation of blood loss by obstetric care providers.

In fact, during the training sessions which recreated postpartum blood loss after a vaginal delivery, the average estimation of the blood loss when using the collector bag and the baby weight scale was 87.3% accurate, according to our primary endpoint which included a 20% margin of error. While using the visual aid depicting known volumes of blood on obstetric materials (in addition to the collector bag and baby scale), the average estimation was 54.1% accurate. For the higher volume (2500 ml) accuracy was 78.3% and for the lower volume (350 ml) accuracy was reduced even further to 33.3%. Our margin of error of 20% concurred with the current literature as it has been commonly used as a percentage of error that is considered significant in other studies evaluating the accuracy in the estimation of blood loss by professionals working in maternity wards while using a visual scale^13^,^22^.

Zuckerwise *et al*.^20^ evaluated the use of a visual aid to improve obstetric care providers’ accuracy in the estimation of postpartum blood loss. In this simulation study on 151 participants, Zuckerwise *et al*. evaluated improvement in estimation of blood loss before and after use of a visual aid. Before using it, estimation of blood loss was only a visual estimation without the use of any other objective tool (such as a collector bag). After, with the use of the visual aid, subjective estimation improvement occurred in 90% of the population, according to the post-test survey. In Zuckerwise *et al*’s study no other objective tool was used in protocol before the use of the visual aid, to estimate blood loss. Therefore, it appeared to them that a visual aid could be useful to help obstetric providers to objectively estimate, postpartum blood loss. However, several studies have shown that visual estimation only of blood loss was inaccurate and lead to underestimations of up to 32%^9^,^10^,^11^,^14^,^15^,^22^,^24^,^25^,^26^,^27^,^28^,^29^. Interestingly, our study showed an increased accuracy using only a collector bag compared to using a visual aid in addition to a collector bag. The use of such a tool for all deliveries could reduce the underestimation of blood loss and thereby avoid under-treatment for women falsely categorized as not having PPH. Use of a baby weight scale to help health care providers on their blood loss estimation should be part of every PPH diagnosis since it is a very reliable easy access tool with a low cost expenses. These results suggest that a visual aid is not useful and could be, moreover, considered detrimental when providers already use an adequate tool such as a collector bag to assess blood loss. Our study does present some limitations. Obstetrics providers were more familiar with the use of the collector bag which is part of our daily protocol in labor wards for the estimation of blood loss, which could explain a better accuracy in the estimation of postpartum blood loss while using it. Using the visual aid in an effective manner may require practice with feedback about how estimation matches actual blood loss. However, as in real life, when a new tool is being used for the first time, a learning period is necessary before becoming familiar with it. In the Zuckerwise *et al*. study^20^ the visual aid was used for the first time as well. Also, this study did not evaluate blood loss, while using the collector bag or the visual aid, during real clinical situations. Thus, there was no maternal outcome improvement rate while using a collector bag and or a visual aid after vaginal deliveries during real clinical situations. Although training simulation sessions are obviously not considered real clinical situations, in our study participants were asked to quickly evaluate blood loss (contained in the collector bag and on different obstetric material) within two minutes to attempt to recreate a more time sensitive clinical environment as in a real clinical situation. For participants to be acting as in a real PPH clinical situation, each training occurred in a delivery room, with artificial blood comparable in color and density to real blood. In addition, each estimation was compared to a known volume, which made our high percentage of accuracy when using the collector bag even more dependable.

## Conclusion

The use of a visual aid in addition to a collector bag does not improve obstetric care providers’ estimation of postpartum blood loss. Its use seems to be detrimental and results in a much more frequent overestimation of estimated blood loss which may lead to unnecessary treatment of PPH.

## Additional Information

**How to cite this article**: Brooks, M. *et al*. Use of a Visual Aid in addition to a Collector Bag to Evaluate Postpartum Blood loss: A Prospective Simulation Study. *Sci. Rep.* **7**, 46333; doi: 10.1038/srep46333 (2017).

**Publisher's note:** Springer Nature remains neutral with regard to jurisdictional claims in published maps and institutional affiliations.

## Figures and Tables

**Table 1:** Population characteristics.

Total population (n = 69)	Participants n (%)
*Profession*	
Obstetricians/Gynecologists	10 (14.5%)
Resident in obstetrics and gynecology (OBGYN)	8 (11.6%)
Midwives	51 (73.9%)
*Age*	
[23–33] years old	41 (59.4%)
[33–43] years old	18 (26.1%)
>43 years old	10 (14.5%)
*Gender*	
Female	58 (84.1%)
Male	11 (15.9%)
*Professional experience in years*	
Less than 5 years	27 (39.1%)
In between 5 to 10 years	22 (31.9%)
More than 10 years	20 (29%)
*Time spent in labor room on an average work shift (in percentage)*	
Less than 25%	10 (14.5%)
25% to 50%	20 (29%)
50% to 75%	37 (53.6%)
More than 75%	2 (2.9%)
*Time spent at the hospital on the day of the training session*	
Less than 12 hours	85 (86.7%)
More than 12 hours	13 (13.3%)
*Professional experience* Professional experience working at Angers’ University Hospital (in years)*	5 (1–8)
Experience using the collector bag (in years)*	4 (2–7)

^*^parameters in median and interquartile range

**Table 2:** Measurement accuracy of postpartum blood loss with and without the use of a visual scale for volumes of 350 ml, 1100 ml, and 2500 ml.

Volume to estimate (ml)	Without using a visual scale			Using a visual scale			p * using wilcoxon test
	Underestimation n (%)	Mean n (%)	Overestimation n (%)	Underestimation n (%)	Mean n (%)	Overestimation n (%)	
350	1 (1.5)	59 (85.5)	9 (13.0)	0 (0.0)	23 (33.3)	46 (66.7)	<0.01
1100	3 (4.3)	61 (88.4)	5 (7.2)	0 (0.0)	35 (50.7)	34 (49.3)	<0.01
2500	5 (7.2)	61 (88.4)	3 (4.3)	2 (2.9)	54 (78.3)	13 (18.8)	<0.01

*p ≤ 0.05 considered as significant.

*p was used to compare the mean of the blood loss estimation for volumes of 350 ml, 1100 ml, and 2500 ml with and without the use of a visual.

Underestimation: estimation <20% of real volume of blood loss.

Overestimation: estimation >20% of real volume of blood loss.
